# Multiple cannulated screw fixation of young femoral neck fractures

**DOI:** 10.12669/pjms.316.8356

**Published:** 2015

**Authors:** Joo Yong Kim, Gyu Min Kong, Dae Hyun Park, Dae Yoo Kim

**Affiliations:** 1 Joo Yong Kim, MD. Dept. of Orthopaedic Surgery, Busan Paik Hospital College of Medicine, Inje University, Bokjiro 75, Busanjin Gu, Busan, 633-165, Korea; 2 Gyu Min Kong, MD. PhD. Dept. of Orthopaedic Surgery, Busan Paik Hospital College of Medicine, Inje University, Bokjiro 75, Busanjin Gu, Busan, 633-165, Korea; 3 Dae Hyun Park, MD. Dept. of Orthopaedic Surgery, Busan Paik Hospital College of Medicine, Inje University, Bokjiro 75, Busanjin Gu, Busan, 633-165, Korea; 4 Dae Yoo Kim MD. Dept. of Orthopaedic Surgery, Busan Paik Hospital College of Medicine, Inje University, Bokjiro 75, Busanjin Gu, Busan, 633-165, Korea

**Keywords:** Femoral neck fracture, Multiple screw fixation, Avascular necrosis, Nonunion

## Abstract

**Objective:**

We wanted to analyze the factors affecting the results of multiple cannulated screws fixation in patients less than 60 years old with femoral neck fracture (FNF).

**Methods:**

We reviewed 52 patients (30 males, 22 females) who were treated with multiple cannulated screws fixation for FNFs. They were followed up for more than one year during January 2002 to December 2012. They were classified by Garden’s classification. The anatomic reduction was evaluated by Garden’s alignment index on hip both anteroposterior and lateral images. Postoperative complications were analyzed during follow up periods.

**Results:**

By Garden’s classification, 6 cases were in stage I, 13 cases in stage II, 30 cases in stage III and 3 cases in stage IV. During follow up periods, avascular necrosis of the femoral head was observed in 12 cases (23%) and nonunion was observed in 5 cases (9%). The 16 patients who had complications underwent total hip arthroplasty (31%). In non-displaced fracture groups (Garde I, II) did not have AVN nor nonunion. The incidence of complications in displaced fracture group was 51.5%. The complicated cases showed tendency for increased apex anterior angulation of femoral neck on hip lateral images and the result was statistically significant. (p=0.0260).

**Conclusion:**

The patients less than 60 years old who were treated with multiple cannulated screws fixation for displaced FNFs showed the incidence of complications was more than 50%. It needs a cautious approach for anatomical reduction, especially related to anterior angulation on hip lateral image.

## INTRODUCTION

Femoral neck fracture (FNF) is likely to develop into conditions that require hip joint replacement even after appropriate treatment due to complications, such as avascular necrosis of the femoral head (AVN) and nonunion.^1^-^3^ Studies have been conducted on various factors related to the high frequency of complications, and they have reported pattern of the fracture, the accuracy of reduction, and the locations of metal implants, the degree of crushing of the posterior cortical bone, and the time interval between being injured and undergoing an operation.^2^,^4^,^5^

Although joint replacement is presented as a good solution for elderly FNF patients as it facilitates early rehabilitation and reduces the risk of reoperation,^6^ techniques that preserve original joints are preferred for relatively younger patients.^7^-^8^ In the present study, the authors followed up with relatively young patients that underwent multiple cannulated screw fixation as a treatment for FNFs to examine the postoperative results and factors that affect complications and prognoses.

**Fig.1:**
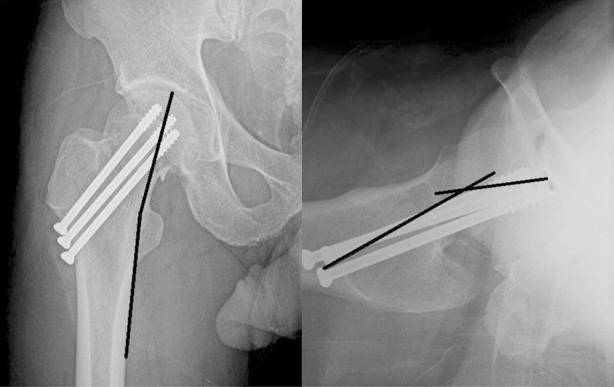
After fixation of femoral neck fracture. We measured trabecular-shaft angle on Anteroposterior radiograph and Anterior angulation on lateral view.

## METHODS

### 1. Study subjects

The present study was conducted with 52 patients (males: 30, females: 22) who could be followed up for at least one year, who were diagnosed with a FNF, and who underwent multiple cannulated screw fixation at our institute between January 2002 and December 2012. The mean age of the subjects at the time of operation was 44.3 years (24–60 years) and the mean follow-up period was 23 months (13–43 months). FNFs were divided into types I, II, III, and IV using the Garden classification.^9^

### 2. Surgical method and rehabilitation

As regards surgical methods, cannulated screws were fixed in all cases and open reduction was not performed in any of the cases. After putting the patient under general anesthesia or spinal anesthesia and laying the patient in a supine position on the fracture table, reduction was performed under C-arm imaging. When the appropriate reduction had been obtained, a point between the greater trochanter and the lesser trochanter was marked on the skin on the lateral side of the femoral region. The region was incised to insert three or four 7.0-mm cannulated screws (AO/ASIF synthes®, 16 or 32 mm thread length), ensuring the threads of the screws completely passed the fracture region to fix the region with compression. A crutch gait and partial weight bearing were allowed at 3–4 weeks after the operation and full weight bearing was allowed at 8–10 weeks after the operation.

### 3. Radiological evaluation

The angle between medial trabecular stream in femoral head and medial cortex of femoral shaft (trabecular-shaft angle) was measured from postoperative hip anteroposterior radiograph and the anteroposterior angulation was measured from lateral image. Classifying the FNF type and measuring the angles were performed by two orthopaedic surgeons reaching a consensus. In patients where there was no gap in the fracture line and where the bony trabeculae were connected to each other across the fracture region in plain radiographs were judged as bone union. Nonunion was defined by the absence of any fracture healing after a period of 6 months.^10^ To evaluate AVN, the radioloreaphic criteria of Ficat and Arlet were used.^11^

### 4. Statistical analysis

SPSS 7.0 was used as a statistical program and the Mann–Whitney U-test was used to judge significance. A *P*-value of ≤0.05 was considered significant.

## RESULTS

**Fig.2:**
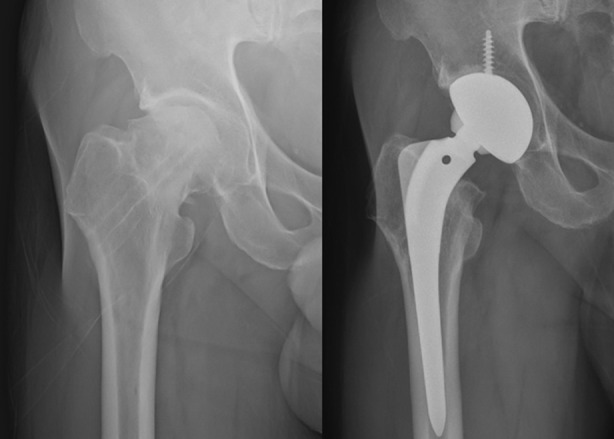
This patient is 63-year-old male. The radiograph taken 22 months after hardware removal show avascular necrosis of right-side femoral head. So we performed total hip replacement arthroplasty.

The subjects comprised 6 type Is, 13 type IIs, 30 type IIIs, and 3 type IVs under the Garden classification method. AVN occurred in 12 cases, and nonunion occurred in 5 cases. Total hip joint replacement was performed due to complications in 16 patients or 31% of the study subjects (Fig.2). When the subjects were divided into two groups, Garden I, II types (non-displaced fracture) and Garden III, IV types (displaced fracture), no cases of AVN or nonunion occurred in the non-displaced fracture group, while there were 17 cases in the displaced fracture group with a complication ratio of 51.5% (Table-I). AVN occurred between 11 months and 23 months after operation, and the average occurrence time was 15.3 months after operation.

**Table-I:** The frequency of AVN and nonunion after primary osteosynthesis for femoralneck fracture according to fracture type and Garden’s classification.

	Classification	Number	AVN	Nonunion	Complication rate
Non-displaced Fx.	Garden I	6	0	0	0%
	Garden II	13	0	0	
Displaced Fx.	Garden III	30	11	3	51.5%
	Garden IV	3	1	2	

The time taken after being injured until operation did not exceed 24 hours in all cases, and there was no difference in operation delay time between patients with complications and those without complications.

Whether or not to perform reduction during operation was determined using the Garden alignment index.^9^ In plain radiography conducted after operation, the degree of anterior angulation of the femoral neck was shown to have considerable effects on operative results. All cases that had AVN or nonunion were displaced fractures in Garden stage III or IV, and the average size of the anterior angulation of the femoral neck was 15.4° (3.3–25.2°). In patients with no complications in Garden stage III or IV, the average size of the anterior angulation of the femoral neck was 9.9° (1.9–21°), and this was statistically significant in comparison to cases with complications (*P*=0.026). However, under anteroposterior radiograph, Trabecular-shaft Angle were shown to have no differences between patients with complications and those without complications (*P*=0.357; Table-II).

**Table-II:** Garden’s alignment index according to presence of complication in displaced group.

Displaced fracture	Complication	Mean angle(°)	P-value
Trabecular-shaft Angle (AP view)	Without Complication	161.87 (140.2 ~ 177.2)	0.357
	With Complication	165.21 (144.1 ~ 179.3)	
Anterior-posterior angulation (Lateral view)	Without Complication	10.29 (3.9 ~ 23.4)	0.026
	With Complication	16.91 (1.9 ~ 39.8)	

## DISCUSSION

Anatomic reduction and internal fixation with an emphasis on preservation of the blood supply to the femoral head is the treatment of choice for younger patients.^2^ However there are high risk of complications associated with FNFs in young patients which result from high-energy trauma.^2^,^12^ The femoral neck is within the joints under the anatomical structures. When a fracture occurs here, complications including avascular necrosis and nonunion frequently occur because the characteristics of blood circulation, and the lack of cambium layer of the periosteum of femoral neck.^13^ Cannulated screw fixation for FNF shows failure rates of approximately 5–30%,^10^,^14^-^15^ and these failure cases mostly require another surgery. The results of the treatment of FNFs are understood to be affected by many factors. Many authors have reported on the degree of displacement of a fracture, the accuracy of reduction, the location of internal fixtures, the degree of crushing of the posterior cortical bone, and the time interval between being injured and undergoing an operation as important factors that affect treatment results.^4^-^5^

Parker^16^ reported that postoperative nonunion occurred in 13% of 470 FNF patients, and of the 13%, 17% were patients with Garden stage III, IV displaced fractures and 8% were patients with Garden stage I, II non-displaced fractures (p<0.005). Therefore, he advised that the preoperative degree of displacement of the fractures was the most important factor for predicting postoperative nonunion, as well as that the classification of fractures was important in determining treatment methods. Although, the Garden classification categorizes fractures according to the degree of displacement of the bone fragments and is considered excellent. However, it has limitations in that it does not consider anatomical classifications that can reflect the fact that the degree of displacement of a fracture does not accurately coincide with radiologic findings in subcapital fractures and the differences in prognoses between subcapital fractures and transverse neck fractures. Moreover it produces larger differences among observers.^17^ In the present study, 17 complications occurred only in the Garden III, IV type fracture group with severe displacements, showing a complication rate of 51.5%. This result indicates that the degree of displacement is related to prognoses and it was consistent with the results of other studies.

Vascularity is important, and it affects the survival of the femoral head. If the bone fragment is rotated during reduction or insertion of the fixatives, blood vessels will be blocked, leading to AVN, which is one of the most common complications of FNFs, the frequency of occurrence of which has been reported as 15-40%.^9^ Avascular necrosis is also affected by the time from fracture to operation, and the preservation of the remaining blood vessels during the time between the initial injury and the internal fixation determines the destiny of the femoral head.^18^-^19^ Manninger et al^19^ advised that fixing the bones within six hours after fracture is important. In our cases, whether or not the time point of the operation was an important element of the occurrence of avascular necrosis could not be identified, because operations in all patients were performed within 24 hours after the patients were injured.

Researchers reported that the accuracy of reduction affects prognoses.^1^,^20^ Nilsson et al^21^ stated that the accurate reduction of fractures during operation is the most important factor for prognoses, because accurate reduction maximizes the contact surface of fractures thereby maximizing blood supply. In the present study, the average size of the anterior angulation of the femoral neck was 15.4° (3.3–25.2°) in cases with complications, while it was 9.9°(1.9–21°)in displaced fracture group without any complications. This difference was statistically significant (P=0.026). The standard for reduction provided by the Garden alignment index recommends not to form angulations of 20° or higher.^9^ However, according to the results of the observations in the present study, even if the degrees of angulation found in imaging examinations conducted during operations were permitted, patients in whom complications occurred showed a tendency to have higher degrees of anterior angulation. Therefore, the likely range of angulation should be reduced further. In our experience, there was a tendencys of under reduction of the femoral neck on the lateral views of C-arm images during operations. The posterior cortical bone tends to be crushed when a FNF has occurred, so it makes the anterior angulation.^9^

### Limitations of the study

It is a simple X-ray based study, so we could not evaluate the FNF three-dimensionally. Zhou et al^22^ reported three dimensional analysis of femoral fractures, and there is a room for application to the FNF. The data of the functional outcome was insufficient. These ware because the present study was a retrospective study wherein operative procedures may be a little different because the patients were operated by numerous surgeons. In addition, although the patients’ bone densities, the locations of the cannulated screws, and the anatomical locations of the fractures, which are important factors of the instability of fractures, are known to affect prognoses, these factors were not analyzed in the present study. Moreover the number of patient was relatively small, and the statistical analysis was not powerful. Therefore, additional prospective studies are necessary.

## CONCLUSION

When multiple-cannulated screw fixation was performed in FNF patients younger than 60 years, complications occurred in at least 50% of patients with Garden III, IV-type fractures, which are displaced fractures. In addition, the state of reduction immediately after operation was judged an important prognostic factor. Therefore, when treating displaced FNFs, the range of reduction should be strictly applied to avoid anterior angulation of the femoral neck during operations, and efforts should be made to obtain accurate anatomical reduction.
